# P-1742. The Role of T2 Candida in the Early Detection of Invasive Candidiasis Among Solid Organ Transplant Patients

**DOI:** 10.1093/ofid/ofaf695.1913

**Published:** 2026-01-11

**Authors:** Megha Jagannathan, Zachary W Hanna, Michael P Veve, Navina K Birk, George J Alangaden, Eloy E Ordaya

**Affiliations:** Henry Ford Hospital, Detroit, MI; Henry Ford Health, Detroit, Michigan; Eugene Applebaum College of Pharmacy and Health Sciences, Detroit, MI; Henry Ford Health System, Detroit, Michigan; Henry Ford Health, Detroit, Michigan; Henry Ford Health, Detroit, Michigan

## Abstract

**Background:**

Invasive Candidiasis (IC) is the leading cause of invasive fungal infections in solid organ transplant recipients (SOTr). T2Candida is a rapid diagnostic test that can improve antifungal use in SOTr. We describe the clinical characteristics and outcomes of SOTr who underwent T2Candida testing (T2) for suspected IC at our institution.

**Table 1.**
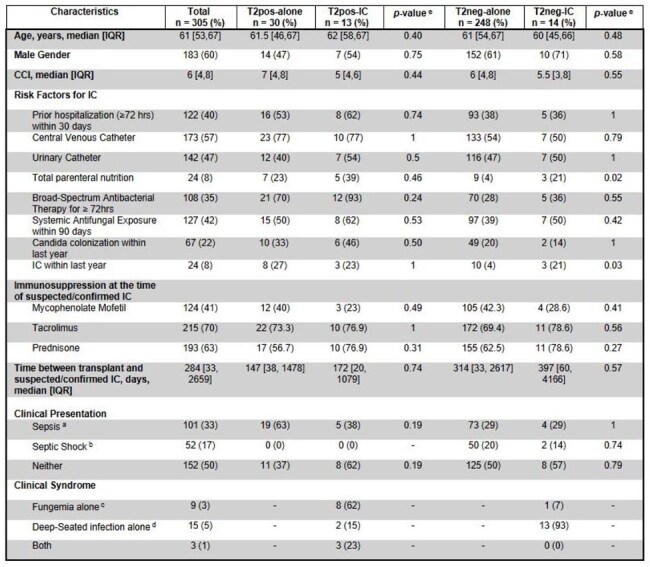
Demographics, risk factors, and clinical presentation of suspected/confirmed IC in SOTr with T2pos-IC vs T2pos-alone and T2neg-IC vs T2neg-alone (n= 305) a Sepsis was classified as 2/4 Systemic Inflammatory Response Syndrome criteria. b Septic Shock was classified as sepsis with hypotension requiring pressor use. c Fungemia alone was classified as patients with positive blood cultures, but no positive sterile tissue cultures for Candida spp. within 48hrs of T2 collection. d Deep-seated infection alone was classified as negative blood cultures, but positive sterile tissue cultures for Candida spp. within 48hrs of T2 collection. e p values calculated by Wilcoxon rank sum tests (continuous variables) and Fischer's Exact test (categorical variables) Abbreviations: CCI, Charlson Comorbidity Index; IQR, Interquartile Range; IC, invasive candidiasis; T2pos-alone, patients with isolated positive T2Candida® Panel without corresponding isolation of Candida spp. on blood/sterile cultures within 48hrs of T2 collection; T2pos-IC, patients with positive T2Candida® Panel with isolation of Candida spp. on blood/sterile tissue cultures within 48hrs of T2 collection; T2neg-alone, patients with negative T2Candida® Panel and negative blood/sterile cultures within 48hrs of T2 collection; T2neg-IC, patients with negative T2Candida® Panel and isolation of Candida spp. on blood/sterile cultures within 48hrs of T2 collection.

**Figure 1.**
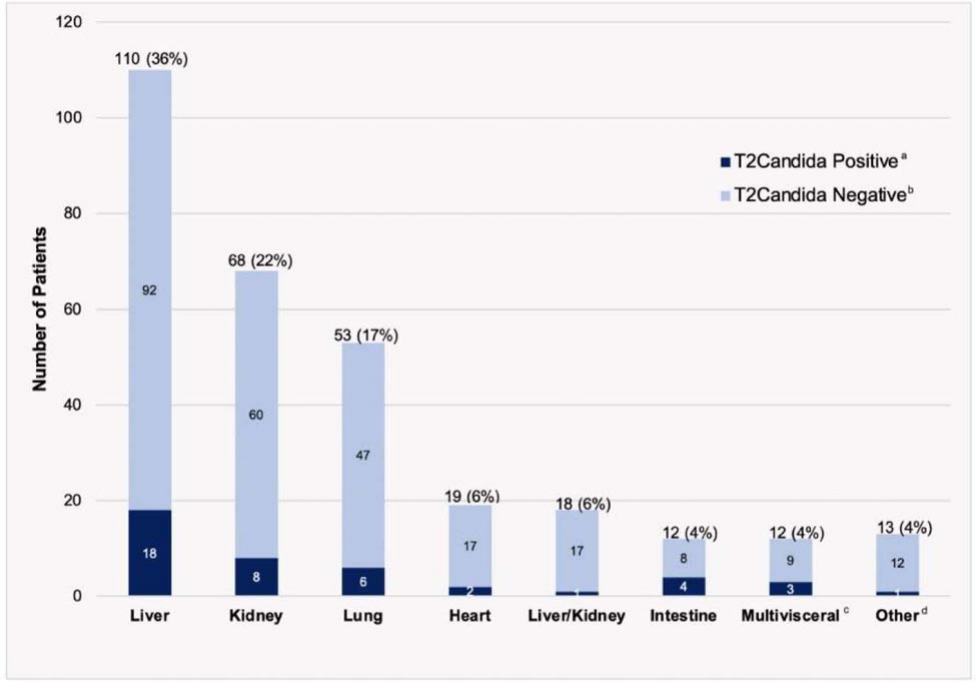
Types of transplanted organs in SOTr who underwent T2Candida® Panel testing (n = 305) a T2Candida Positive, noted in dark blue, refers to any patient with a positive T2Candida® Panel. b T2 Candida Negative, noted in light blue, refers to any patient with a negativeT2Candida® Panel. c Other refers to patients with other combinations of organ transplants (e.g. kidney/pancreas was most frequent). d Multivisceral includes patients with ≥ 3 abdominal organs transplanted. Abbreviations: SOTr, Solid organ transplant recipients

**Methods:**

A retrospective study including adult SOTr who underwent T2 for suspected IC between 01/2015-12/2024. We compared data of patients with positive T2 alone (T2pos-alone) vs. positive T2/positive culture from sterile site (T2pos-IC), and patients with negative T2 (T2neg-alone) vs. negative T2/positive culture (T2neg-IC).

**Figure 2.**
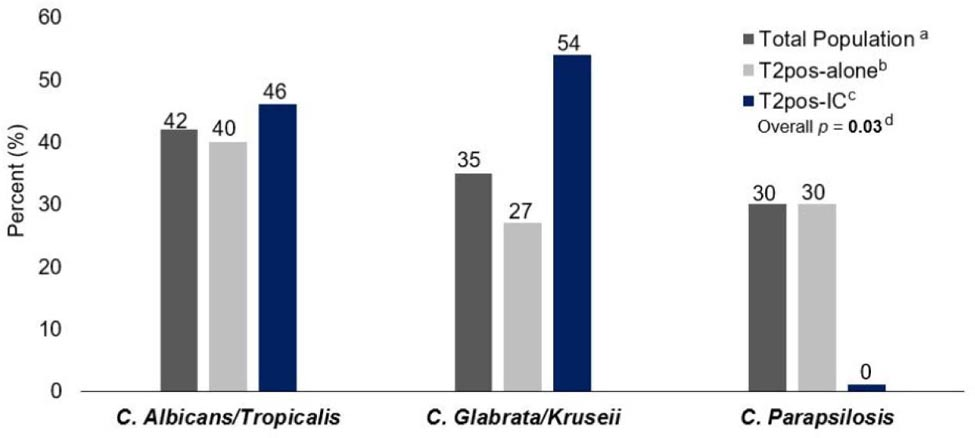
Candida species detected by T2Candida® Panel in SOTr with T2pos-alone and T2pos-IC (n=43) a Total population, all patients with a positive T2Candida® Panel, denoted in dark grey. b T2pos-alone, patients with isolated positive T2Candida® Panel without corresponding isolation of Candida spp. on blood/sterile cultures within 48hrs of T2 collection, denoted in light grey. c T2pos-IC, patients with positive T2Candida® Panel with isolation of Candida spp. on blood/sterile tissue cultures within 48hrs of T2 collection, denoted in dark blue. d p values calculated via Fischer's Exact test. Abbreviations: SOTr, Solid organ transplant recipients; C, Candida; IC, invasive candidiasis

**Table 2.**
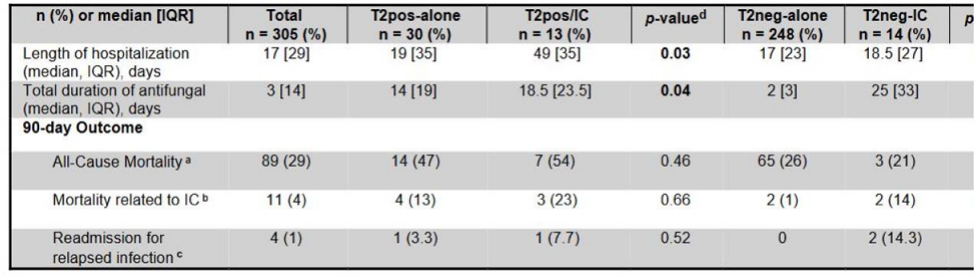
Clinical Outcomes of SOTr with T2pos-IC vs T2pos-alone and T2neg-IC vs T2neg-alone (n = 305) a All-cause mortality refers to death from any cause. b Mortality related to IC refers to death because of documented sepsis secondary to Candida spp. identified on sterile cultures. c Readmission for relapsed infection refers to patients hospitalized for worsened documented infectious signs or symptoms via clinical evaluation or imaging studies demonstrating worsened deep-seated infection. d p values calculated via Wilcoxon rank sum tests (continuous variables) and Fischer's Exact test (categorical variables) Abbreviations: IQR, Interquartile Range; IC, invasive candidiasis; T2pos-alone, patients with isolated positive T2Candida® Panel without corresponding isolation of Candida spp. on blood/sterile cultures within 48hrs of T2 collection; T2pos-IC, patients with positive T2Candida® Panel with isolation of Candida spp. on blood/sterile tissue cultures within 48hrs of T2 collection; T2neg-alone, patients with negative T2Candida® Panel and negative blood/sterile cultures within 48hrs of T2 collection; T2neg-IC, patients with negative T2Candida® Panel and isolation of Candida spp. on blood/sterile cultures within 48hrs of T2 collection.

**Results:**

From the 305 SOTr included, 43 (14%) had a positive T2. Baseline characteristics were comparable between groups (Table 1). Median time from transplant to suspected/confirmed IC was 284 [33-2659] days. Transplanted organs included liver (36%), kidney (22%), and lung (17%) (Fig 1). In SOTr with positive T2, 13 (30%) had concomitant isolation of Candida spp. in cultures (T2pos-IC) (Fig 2). Median duration of hospitalization and antifungal therapy (AFT) was significantly shorter in the T2pos-alone group vs. T2pos-IC (19 vs. 49 days, *p*=0.03, 14 vs. 19 days, *p*=0.04, respectively). 90-day all-cause mortality was highest in T2pos-IC (Table 2). In SOTr with negative T2, 248 (95%) did not have concomitant isolation of Candida spp. in cultures (T2neg-alone). Based on T2 results, 64 (24%) SOTr stopped AFT in ≤48hrs and 145 (55%) did not receive any AFT. Median duration of AFT, IC-related 90-day mortality, and readmission due to relapsed infection were significantly lower in the T2neg-alone group compared to T2Neg-IC (2 vs. 25 days, *p*< 0.01, 1% vs 14% *p*=0.02, 0 vs 14% *p*< 0.01, respectively) (Table 2).

**Conclusion:**

SOTr with T2pos-IC had higher mortality and significantly higher duration of hospitalization and AFT compared to T2pos-alone. A negative T2 frequently prompted discontinuation or avoidance of AFT. Patients with T2neg-alone had low mortality and no readmission for suspected IC. Further studies are needed to evaluate the role of rapid diagnostics in the SOTr population.

**Disclosures:**

All Authors: No reported disclosures

